# Evaluation of the feasibility, safety, and preliminary effectiveness of hyperthermic intraperitoneal chemotherapy following radical surgery for locally advanced gastric cancer

**DOI:** 10.3389/fonc.2025.1498388

**Published:** 2025-09-09

**Authors:** Tianze Zhang, Hang Yu, Lang Wang, Shijun Zhao, Cheng Zhao, Jie Chai, Dehai Wang

**Affiliations:** ^1^ Department of Gastrointestinal Surgery The Second Hospital, Cheeloo College of Medicine, Shandong University, Jinan, China; ^2^ Department of Gastrointestinal Surgery, Shandong Cancer Hospital and Institute, Shandong First Medical University & Shandong Academy of Medical Science, Jinan, Shandong, China

**Keywords:** intraperitoneal hyperthermic chemotherapy, gastric cancer, advanced stage, survival analysis, peritoneal metastasis recurrence

## Abstract

**Objective:**

To explore the feasibility, safety, and potential association between hyperthermic intraperitoneal chemotherapy (HIPEC) and peritoneal recurrence in patients with locally advanced gastric cancer following D2 radical surgery.

**Materials and methods:**

A retrospective analysis was conducted on 156 patients with locally advanced gastric cancer treated with D2 surgery at two centers between 2014 and 2023. Clinical outcomes and adverse events were assessed.

**Results:**

Baseline characteristics were comparable between the HIPEC group (n = 70) and the surgery-only group (n = 86). The 2-year peritoneal recurrence rate was lower in the HIPEC group (18.6% vs. 34.9%, *P*=0.0206). Several factors—including high Charlson Comorbidity Index, advanced T stage, vascular invasion, intraoperative blood loss, and absence of HIPEC—were associated with higher recurrence risk. No significant differences were observed in perioperative complications between the groups or among different HIPEC frequencies (all *P* > 0.05).

**Conclusion:**

In this real-world dual-center study, HIPEC following D2 surgery was found to be feasible and safe, and was associated with a reduced risk of peritoneal recurrence in patients with locally advanced gastric cancer. These observational findings warrant further validation in randomized controlled trials.

## Introduction

Gastric cancer remains a major global health burden, ranking as the fifth most common cancer and the fourth leading cause of cancer-related deaths worldwide (1). Current treatment options for gastric cancer include surgery, chemotherapy, targeted therapy, radiation therapy, and immunotherapy (2–5). However, in recent years, these treatments have shown limited clinical progress, with a median survival of only about 8 months for gastric cancer (6). Currently, the first-line treatment for locally advanced gastric cancer includes D2 radical surgery combined with systemic chemotherapy, according to major guidelines, both postoperative adjuvant chemotherapy and perioperative systemic therapy are recommended, depending on clinical stage and patient condition (7). It has been reported that perioperative systemic chemotherapy for locally advanced gastric cancer patients undergoing D2 radical surgery can significantly prolong patients’ survival (8).

Peritoneal metastasis is a major form of locally advanced gastric cancer, and for potentially curable gastric cancer patients, the peritoneum is one of the most common sites of recurrence (9). Peritoneal metastasis can lead to refractory ascites, progressive intestinal obstruction, and severe abdominal pain, significantly affecting patients’ overall survival and quality of life. Currently, there are no effective treatments to prevent the recurrence of peritoneal metastasis. Even for locally advanced gastric cancer patients undergoing D2 radical surgery combined with postoperative systemic chemotherapy, the postoperative peritoneal metastasis rate and 5-year mortality remain high (6, 10). Currently, cytoreductive surgery (CRS) combined with hyperthermic intraperitoneal chemotherapy (HIPEC) has become the main treatment for ovarian cancer, appendiceal mucinous neoplasms, pseudomyxoma peritonei of colonic origin (PMP), mesothelioma, and peritoneal metastasis (11–15). Prospective local-regional treatment using cytoreductive surgery and hyperthermic intraperitoneal chemotherapy has multiple advantages, including the significant removal of tumor burden through surgical resection and localized hyperthermic chemotherapy aiding in eradicating microscopic metastases and invisible free tumor cells (16).

As early as the 1990s, scholars initiated clinical studies to explore the potential effectiveness and safety of prophylactic hyperthermic intraperitoneal chemotherapy (HIPEC) following surgery for gastric cancer (17, 18). However, the early evidence was limited by suboptimal study designs and immature technical platforms. In recent years, with improvements in HIPEC-related theory, equipment, and implementation, there has been renewed interest in its clinical value. To further investigate the feasibility, safety, and potential association between postoperative prophylactic HIPEC and peritoneal recurrence, we conducted a dual-center retrospective study based on real-world data from patients with locally advanced gastric cancer treated at the Second Hospital of Shandong University and Shandong Cancer Hospital.

## Materials and methods

### Study population and grouping

Using a descriptive case series research method, clinical and pathological data of locally advanced gastric cancer patients who underwent D2 radical surgery (with or without HIPEC) at the Second Hospital of Shandong University and Shandong Cancer Hospital, affiliated with the First Medical University of Shandong Province, from January 2014 to February 2023 were retrospectively collected.

The inclusion criteria were as follows:(1) Age between 18 and 80 years;(2) Postoperative pathological confirmation of locally advanced gastric cancer;(3) No evidence of distant metastases detected on preoperative imaging;(4) Underwent D2 radical resection with R0 margins achieved;(5) Patients in the HIPEC group received at least one session of HIPEC;(6) No prior history of chemotherapy, radiotherapy, or immunotherapy;(7) No contraindications to chemotherapy based on laboratory tests, electrocardiogram (ECG), or comorbidities.

The exclusion criteria were as follows:(1) Incomplete clinicopathological data during the perioperative period or loss to follow-up;(2) Intraoperative identification of tumor metastasis;(3) Presence of other concurrent malignancies;(4) History of other malignant diseases;(5) Positive surgical resection margins;(6) Patients who did not receive adjuvant systemic chemotherapy, including those who failed to receive a standard regimen;(7) Postoperative survival time of less than two months.

All clinical decisions, including the use of HIPEC and adjuvant chemotherapy, were made by a multidisciplinary team (MDT) based on individual patient condition, tumor characteristics, and shared decision-making factors. This reflects real-world clinical practice, where therapeutic choices are often tailored rather than protocol-driven. However, the absence of explicitly standardized selection criteria may affect reproducibility and generalizability of the findings.

This study included a total of 156 patients, of which 70 patients were in the HIPEC group, receiving preventive HIPEC combined with postoperative adjuvant chemotherapy after D2 radical surgery. The remaining 86 patients were in the control group and received only postoperative adjuvant chemotherapy without preventive HIPEC. All patients received adjuvant chemotherapy with oxaliplatin and capecitabine (SOX regimen) within 6 weeks after surgery. Clinical and pathological data of all patients were collected.

Specific SOX Treatment Regimen for Gastric Cancer: Day 1 (D1): Oxaliplatin (130 mg/m²): Administered intravenously. D1–D14: S-1 (oral administration): Taken twice daily (once in the morning and once in the evening). Dose adjustment based on body surface area (BSA): BSA <1.25 m²: 40 mg per dose; 1.25–1.50 m²: 50 mg per dose; ≥1.50 m²: 60 mg per dose. D15–D21: Discontinue S-1. Treatment Cycle: Each chemotherapy cycle lasts 21 days, and patients receive a total of 6 cycles of the SOX regimen.

Tumor staging was determined by preoperative imaging, intraoperative findings, or pathological reports. The conduct of this study adhered to the ethical requirements outlined in the Helsinki Declaration, and it received approval from the hospital’s ethics committee with approval number: KYLL2024340, SDTHEC2024003170.

### Treatment method

The HIPEC procedure was conducted as follows: Patients underwent exploratory laparotomy, laparoscopy, or robot-assisted radical gastrectomy under general anesthesia. During the surgery, four drainage tubes were placed in the abdominal cavity: two on both sides of the pelvis, one in the splenic fossa, and one on the hepatic diaphragmatic surface, each with multiple side holes. These tubes were respectively fixed to the right lower abdomen, left lower abdomen, right upper abdomen, and left upper abdomen. The tubes in the splenic fossa and on the hepatic diaphragmatic surface were used for fluid inflow, while those in the pelvic cavity were used for fluid outflow. The first HIPEC treatment was performed immediately after surgery, followed by subsequent abdominal closed HIPEC from postoperative day 2 to postoperative day 7. The second HIPEC treatment was administered 48 hours after surgery, depending on the patient’s postoperative recovery status. The third treatment was contingent upon the patient’s tolerance and willingness to receive therapy, with each treatment spaced 48 hours apart. Fentanyl and bupivacaine were used to relieve pain during HIPEC. HIPEC was performed in a regular ward. Isotonic dialysis fluid (4000 ml) was pumped into the abdominal cavity using an automatic pump at a perfusion rate of 600 ml/min and a temperature of 42-43 °C (monitored and controlled by the HIPEC machine). Perfusion time was 60 minutes to ensure uniform distribution of the perfusate, followed by fluid removal after perfusion. Postoperative hydration included intravenous fluid maintenance, and urine output was monitored according to standard treatment protocols. After the completion of HIPEC, the drainage tubes were retained for 2–3 days to assist in draining residual fluids from the abdominal cavity. Antitumor agents for HIPEC were selected based on the *Expert Consensus on Clinical Application of Intraperitoneal Hyperthermic Chemotherapy in China (2019 edition)*. The key parameters of the HIPEC procedure were as follows: 1. Chemotherapeutic agents: Drugs such as cisplatin, paclitaxel, and oxaliplatin were used, with dosages adjusted according to body surface area and standard systemic chemotherapy reference guidelines. 2. Temperature: The perfusate was maintained at (43 ± 0.1)°C. 3. Duration: Each infusion lasted 60 to 90 minutes, with 60 minutes being the most common. 4. Number of cycles: For patients undergoing multiple C-HIPEC sessions, treatments were administered at 24-hour intervals, with a total of 1 to 3 sessions.

### Evaluation criteria

(1) Evaluation of patients’ preoperative medical history using the Charlson Comorbidity Index (CCI) (19);(2) Evaluation of gastric cancer patients using the American Joint Committee on Cancer (AJCC) TNM staging system, including assessment of tumor infiltration depth, lymph node metastasis, and presence of distant metastasis;(3) Evaluation criteria for adverse events. Adverse reactions refer to any adverse or unexpected signs (including abnormal laboratory findings), symptoms, or diseases temporally associated with medical treatment or procedures, regardless of whether they are considered related to medical treatment or management. Adverse event recording: Two associate chief physicians recorded adverse events of grade 2 or above occurring during the patient’s treatment observation period based on the Common Terminology Criteria for Adverse Events (CTCAE 5.0) published by the U.S. Department of Health and Human Services. These events include hypoalbuminemia, bone marrow suppression, incision-related complications, intra-abdominal infection, pulmonary infection, gastric emptying disorder, anemia, postoperative bleeding, anastomotic leakage, intestinal obstruction, abdominal distension, and liver function impairment. Finally, a senior chief physician reviewed the above adverse events and made a safety assessment of the patients.

### Data collection

Data collection was conducted by retrieving patient information from the medical record retrieval system, laboratory information system, and pathology system of the Second Hospital of Shandong University and Shandong Cancer Hospital, affiliated with the First Medical University of Shandong Province. Data were collected and compiled using Excel spreadsheets. The collected indicators mainly included the following aspects: (1) General patient information, including age, gender, body mass index (BMI), medical history, and ASA classification; (2) Preoperative laboratory indicators, including albumin, hemoglobin, aspartate aminotransferase (AST), and alanine aminotransferase (ALT); (3) Postoperative pathological indicators, including tumor maximum diameter, tumor location, tumor differentiation, presence of neural invasion, presence of vascular invasion, lymph node metastasis status, tumor infiltration depth, and Her2 status; (4) Perioperative adverse events; (5) Relationship between the number of HIPEC treatments and perioperative adverse events.

### Outcome evaluation

postoperative follow-up data for each patient were collected and compiled through outpatient reviews or telephone follow-ups. According to the research objectives of this study, the follow-up endpoint was determined to be March 1, 2024. Patients were followed up every 3 months for the first 2 years postoperatively, and then every 6 months for the next 3 years postoperatively. After 5 years postoperatively, patients were followed up annually.

Abdominal thin-layer enhanced CT (abdominal + pelvic) was determined to be the preferred imaging modality for detecting peritoneal metastasis in gastric cancer. Typical manifestations of gastric cancer peritoneal metastasis include uneven thickening of the peritoneum, high enhancement or nodules, nodular thickening of the mesentery, as well as significant accumulation of fluid in the abdominal and pelvic cavities, along with indirect signs such as dilation of the bile ducts, ureters, and intestines.

### Statistical analysis

Statistical analysis was conducted using IBM SPSS Statistics v25.0 software. For normally distributed continuous data, the mean ± standard deviation ( $\bar{x}$ ±*s*) was used, and intergroup comparisons were performed using the t-test. Skewed distributed continuous data were represented as median (P25, P75) or median (interquartile range, IQR), and intergroup comparisons were conducted using the Mann-Whitney U test. Categorical data were presented as frequencies (percentages), and intergroup comparisons were performed using the chi-square test or Fisher’s exact test. Kaplan-Meier curves were employed to represent survival data, and Cox regression analysis was used to evaluate the impact of various clinical factors on postoperative peritoneal metastasis recurrence. Logistic regression models were utilized for univariate and multivariate analysis of adverse events in patients with locally advanced gastric cancer. Factors with *P*<0.20 in univariate analysis were included in multivariate analysis, and factors with P<0.05 in multivariate analysis were considered independent influencing factors. A two-tailed *P* value <0.05 was considered statistically significant.

## Results

### Patient characteristics

A total of 156 patients met our inclusion and exclusion criteria. Among these patients, 70 were in the HIPEC group, and 86 were in the control group. The two groups were well balanced in terms of age, gender, ASA score, body mass index, Charlson comorbidity index, tumor location, tumor maximum diameter, tumor differentiation, pathological T stage, neural invasion, clinical tumor stage, vascular invasion, intraoperative blood loss, Her2 gene status, postoperative hospital stay, time to oral intake postoperatively, preoperative albumin level, preoperative hemoglobin level, preoperative aspartate aminotransferase level, and preoperative alanine aminotransferase level (**Table 1**).

**Table 1:** Baseline clinical characteristics of patients.

Characteristics	Control group (n=86)	HIPEC group (n=70)	*P-*value
Sex (n, %)			
Male Female	66 (76.7%) 20 (23.3%)	56 (80%) 14 (20%)	0.624
Mean age	64.5 (57.8-70.3)	63.0 (54.8-69.0)	0.831
BMI	22.0 (20.1-24.2)	19.2 (21.2-23.5)	0.281
CCI			
0 1 2 ≥3	40 (46.5%) 24 (27.9%) 10 (12.2%) 12 (14.0%)	32 (45.7%) 19 (27.1%) 9 (12.9%) 10 (14.3%)	0.996
ASA rating			
1 2 3	10 (11.6%) 60 (69.8%) 16 (18.6%)	7 (10.0%) 56 (80.0%) 7 (10.0%)	0.76
Tumor location			
Upper stomach Middle stomach Lower stomach Whole stomach	9 (10.5%) 7 (8.1%) 58 (67.4%) 12 (14%)	10 (14.3%) 14 (20.0%) 35 (50.0%) 11 (15.7%)	0.088
Maximum Tumor Diameter	5.0 (4.0-7.0)	5.5 (4.0-7.1)	0.512
Tumor differentiation grade			
Poorly differentiated Moderately differentiated Well differentiated Signet-ring cell carcinoma	60 (69.8%) 17 (19.8%) 0 (0.0%) 9 (10.5%)	41 (58.6%) 17 (24.3%) 2 (2.9%) 10 (14.3%)	0.258
Neural invasion			
Yes No	53 (61.6%) 33 (38.4%)	45 (64.3%) 25 (35.7%)	0.733
Vascular invasion			
Yes No	47 (54.7%) 39 (45.3%)	40 (57.1%) 30 (42.9%)	0.755
Lymph node metastasis			
N0 N1 N2 N3a N3b	17 (19.8%) 25 (29.1%) 16 (18.6%) 19 (22.1%) 9 (10.5%)	10 (14.3%) 17 (24.3%) 14 (20.0%) 20 (28.6%) 9 (12.9%)	0.758
Invasion depth			
T3 T4a T4b	25 (29.1%) 38 (44.2%) 23 (26.7%)	26 (37.1%) 26 (37.1%) 18 (25.7%)	0.535
Clinical tumor stage			
IIA IIB IIIA IIIB IIIC	3 (4.3%) 11 (15.7%) 13 (18.6%) 20 (28.6%) 23 (32.9%)	10 (11.6%) 15 (17.4%) 15 (17.4%) 13 (15.1%) 33 (38.4%)	0.183 0.670
Preoperative Albumin Level	39.6 (35.6-42.0)	40.3 (35.6-43.1)	
Preoperative Hemoglobin Level	115.8±21.6	117.2±16.0	0.122
Preoperative ALT Level	16.3 (11.0-30.3)	15.5 (10.0-29.0)	0.746
Preoperative AST Level	19.0 (15.0-28.8)	20.2 (16.0-32.4)	0.096
Surgical method			
Open surgery Laparoscopic surgery Robotic surgery	39 (45.3%) 35 (40.7%) 12 (14.0%)	31 (44.3%) 31 (44.3%) 8 (11.4%)	0.853
Intraoperative Blood Loss	65.0 (50.0-100.0)	100.0 (50.0-162.5)	0.179
Her2			
Positive Negative	32 (37.2%) 54 (62.8%)	21 (30.0%) 49 (70.0%)	0.344
Postoperative Hospital Stay	12.0 (9.0-14.0)	13.0 (11.0-18.0)	0.142
Postoperative Feeding Time	6.5 (6.0-8.3)	7.0 (6.0-8.0)	0.070

BMI, Body Mass Index; CCI, Charlson Comorbidity Index; ASA, American Society of Anesthesiologists.

### Peritoneal recurrence analysis

**Figure 1:**
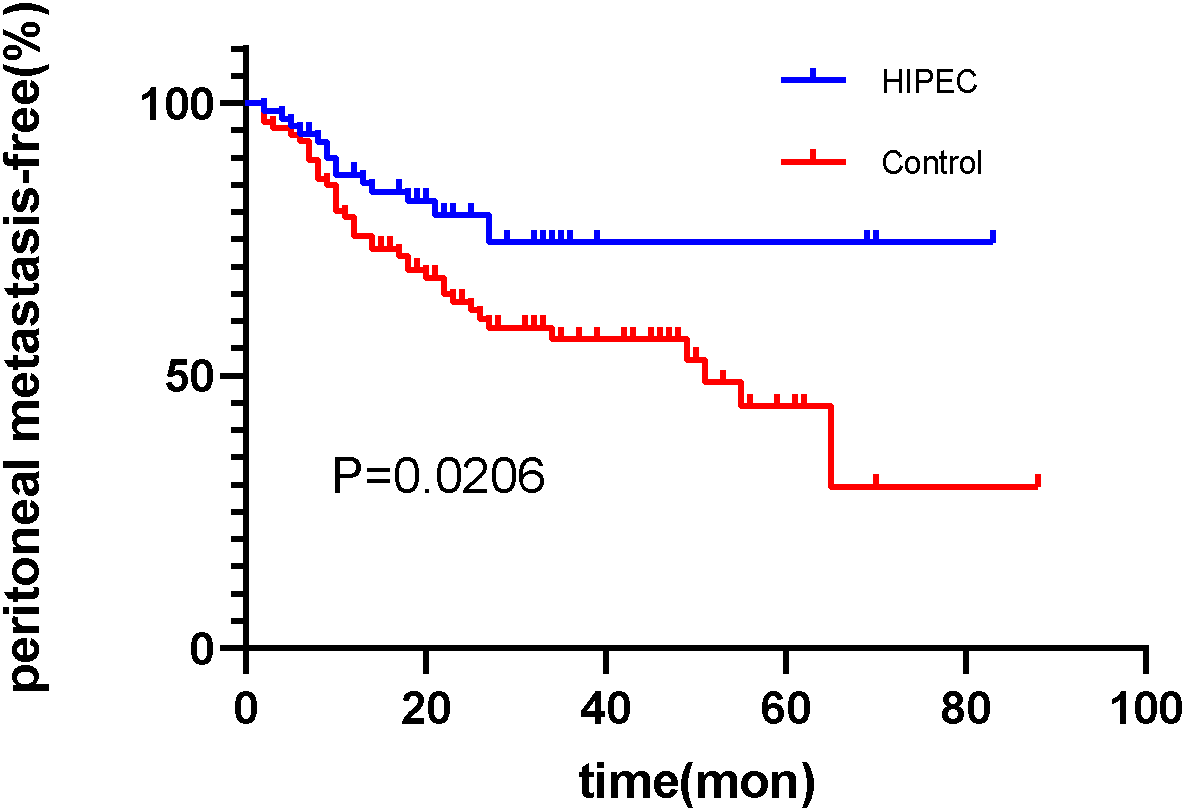
The survival analysis function of peritoneal recurrence after surgery in the HIPEC group and the control group. (Log-rank test, *P*=0.0206).

Through outpatient reviews, telephone inquiries, and other methods, follow-up was conducted for discharged patients. Among the 156 patients, the follow-up duration exceeded 1 year (12 months) for all. In the HIPEC group, the shortest time to peritoneal recurrence after surgery was 2.4 months, with a median time to peritoneal recurrence of 65.3 months (range: 56.6–74.1 months). In the control group, the shortest time to peritoneal recurrence after surgery was 2.1 months, with a median time to peritoneal recurrence of 51.0 months (range: 26.9–75.1 months). The rates of peritoneal recurrence at 1 year and 2 years were 12.9% (9/70) and 18.6% (12/70) in the HIPEC group, respectively, and 20.9% (18/86) and 34.9% (30/86) in the control group, respectively. The difference in postoperative peritoneal recurrence between the two groups was statistically significant (log-rank, *P*=0.0206) (**Figure 1**).

**Figure 2:**
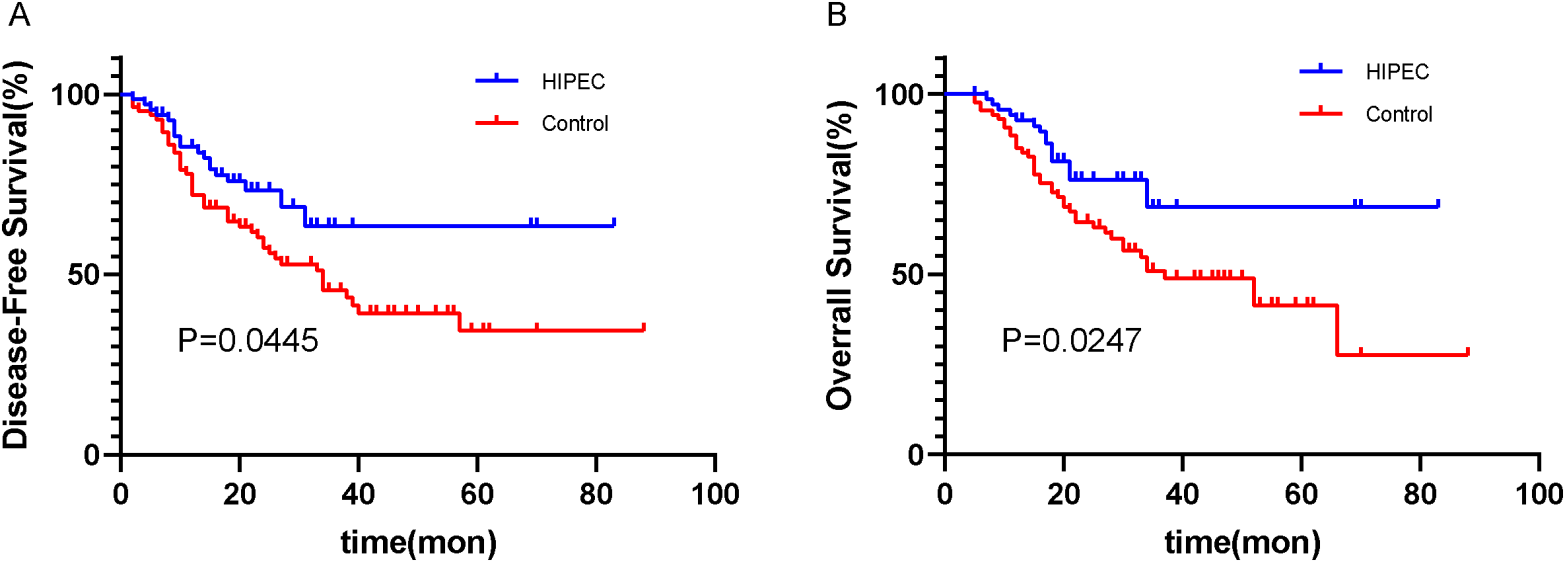
Kaplan–Meier plot of disease-free survival **(A)** and overall survival **(B)** for the patients.

The median follow-up time for data analysis was 20.0 months (range: 12.0–33.0 months). All patients received adjuvant chemotherapy (oxaliplatin + capecitabine for 6 cycles) after surgery. Among the two groups, the disease-free survival (DFS) rate in the HIPEC group was 72.9% (51/70), compared to 46.5% (40/86) in the control group (**Figure 2A**). Furthermore, the overall survival (OS) rate was 78.6% (55/70) in the HIPEC group and 52.3% (45/86) in the control group (**Figure 2B**). The differences in both disease-free survival (DFS) and overall survival (OS) between the two groups were statistically significant, with *P*=0.0445 for DFS and *P*=0.0247 for OS, respectively (**Figure 2**).

We conducted univariate and multivariate Cox regression analyses to determine the factors influencing postoperative peritoneal recurrence. The results showed that a higher Charlson comorbidity index score, higher T stage with greater tumor infiltration, positive vascular invasion, absence of hyperthermic intraperitoneal chemotherapy (HIPEC) treatment, and greater intraoperative blood loss were independent risk factors for peritoneal recurrence after D2 radical resection in patients with locally advanced gastric cancer (**Table 2**).

**Table 2:** Univariate and multivariate Cox analyses of postoperative recurrence in patients with locally advanced gastric cancer in the HIPEC group and the pure surgery group.

Characteristics	Median time to peritoneal metastasis (95% CI)	Hazard ratio for peritoneal metastasis (95% CI)	Univariate analysis (*P*-value)	Multivariate analysis (*P*-value)
Sex (n, %)				
Male Female	55.7 (47.6-63.9) * 47.8 (37.8-57.7) *	1.085 (0.569-2.070) 1	0.805	–
Mean age		0.986 (0.961-1.011)	0.269	–
BMI		0.955 (0.864-1.056)	0.369	–
CCI				
0 1 2 ≥3	57.9 (48.0-67.8) * 53.0 (44.2-61.8) * 18.0 (14.4-46.3) 51.0 (32.4-114.5)	0.639 (0.308-1.329) 0.515 (0.222-1.196) 1.523 (0.683-3.393) 1	**0.040**	**0.001**
ASA rating				
1 2 3	72.4 (56.8-88.1) * 65.0 (12.2-89.1) 35.9 (25.3-46.4) *	0.283 (0.077-1.038) 0.638 (0.318-1.282) 1	**0.145**	0.721
Tumor location				
Upper stomach Middle stomach Lower stomach Whole stomach	59.4 (41.1-77.7) * 53.3 (35.8-70.7) * 48.7 (42.5-54.9) * 26.0 (23.9-28.1)	0.598 (0.211-1.694) 0.748 (0.277-2.022) 0.682 (0.321-1.450) 1	0.743	–
Maximum Tumor Diameter		1.130 (1.037-1.233)	0.005	0.194
Tumor differentiation grade				
Poorly differentiated Moderately differentiated Well differentiated Signet-ring cell carcinoma	51.0 (28.3-78.7) 63.0 (56.6-69.4) * - 27.2 (19.8-34.7) *	0.684 (0.329-1.421) 0.180 (0.055-0.589) (0.000-2.898E+277) 1	**0.041**	0.055
Neural invasion				
Yes No	52.4 (43.5-61.3) * 65.0 (40.6-89.4)		**0.081**	0.812
Vascular invasion				
Yes No	34.0 (11.3-56.2) 68.3 (58.7-77.9) *		**0.002**	**0.020**
HIPEC treatment				
Yes No	65.4 (56.6-74.1) * 51.0 (25.9-75.1)		**0.024**	**0.034**
Lymph node metastasis				
N0 N1 N2 N3a N3b	65.6 (53.4-77.8) * 49.0 (39.4-58.6) * 55.0 (45.9-64.1) 65.0 (22.1-108.3) 23.0 (18.1-27.9)		**0.110**	0.098
Invasion depth				
T3 T4a T4b	65.0 (28.5-120.8) 57.2 (47.8-66.6) * 22.0 (5.6-32.9)	0.239 (0.111-0.515) 0.387 (0.211-0.711) 1	**0.000**	**0.001**
Clinical tumor stage				
IIA IIB IIIA IIIB IIIC	50.4 (39.7-61.1) * 65.1 (52.5-77.7) * 55.2 (44.7-65.8) * 44.1 (34.2-54.0) 46.6 (34.7-58.4)	0.210 (0.043-1.034) 0.346 (0.121-0.992) 0.315 (0.111-0.894) 0.659 (0.273-1.594) 1	**0.047**	**0.017**
Preoperative Hemoglobin Level		0.990 (0.977-1.003)	0.135	0.341
Preoperative ALT Level		1.004 (0.992-1.015)	0.538	–
Preoperative AST Level		1.002 (0.988-1.016)	0.790	–
Surgical method				
Open surgery Laparoscopic surgery Robotic surgery	54.5 (44.8-64.2) * 55.0 (44.9-65.1) 32.1 (28.2-36.1) *	2.560 (0.772-8.487) 2.257 (0.684-7.554) 1	0.305	–
Intraoperative Blood Loss		1.003 (1.001-1.006)	**0.006**	**0.003**
Her2				
Positive Negative	52.1 (42.0-62.2) * 65.0 (11.4-87.3)	1.202 (0.689-2.09) 1	0.517	
Postoperative Hospital Stay		1.056 (1.026-1.087)	0.000	0.909
Postoperative Feeding Time		1.063 (1.025-1.103)	0.001	0.385

*Less than 50% of patients relapsed, and the median time to peritoneal metastasis was absent. The mean (95% CI) time to peritoneal metastasis was used instead.

The bold values represent the results of univariate or multivariate analysis. Factors with P<0.20 in univariate analysis were included in the multivariate analysis, and those with P<0.05 in multivariate analysis were considered statistically significant and independent influencing factors.

### Complications

Adverse events occurring during the perioperative period were evaluated according to the Common Terminology Criteria for Adverse Events (CTCAE) published by the U.S. Department of Health and Human Services. Only events of grade 2 or higher were recorded.

In the HIPEC group, 23 patients (32.9%) experienced a total of 37 adverse events, with the most common being postoperative hypoalbuminemia (12/70, 17.1%), pulmonary infections (6/70, 8.6%), and postoperative bleeding (3/70, 4.3%). Of these, the majority were grade 2 in severity; however, there were 5 grade 3 events, including severe hypoalbuminemia (n=2), postoperative bleeding requiring intervention (n=2), and myelosuppression (n=1). Some complications, such as mild peritoneal irritation or transient myelosuppression, were considered potentially related to the HIPEC procedure.

In the control group, 44 patients (51.2%) experienced 58 adverse events, with the most frequent being postoperative hypoalbuminemia (15/86, 17.4%), anemia (11/86, 12.8%), and pulmonary infections (8/86, 9.3%). The control group had 6 grade 3 adverse events, including severe anemia (n=2), intra-abdominal infections requiring intravenous antibiotics (n=2), and anastomotic leakage requiring intervention (n=2).

All adverse events in both groups were managed conservatively, and symptoms were relieved in most cases. Statistical analysis revealed no significant differences in the incidence of individual adverse events between the HIPEC and control groups (all *P >*0.05). A detailed breakdown of the types, frequency, and distribution of adverse events is presented in **Table 3**.

**Table 3:** Perioperative adverse events (Grade ≥2) in the HIPEC group and the control group.

Perioperative period Adverse events	Control group (n=86)	HIPEC group (n=70)	Grade 3 events (Control/HIPEC)	*P-*value
Hypoalbuminemia	15	12	2/2	0.961
Myelosuppression	0	2	0/1	0.389
Incisional complications	3	0	1/0	0.321
Intra-abdominal infection	8	2	2/1	0.192
Pulmonary infection	8	6	1/1	0.874
Delayed gastric emptying	0	0	0/0	–
Anemia	11	4	2/1	0.136
Postoperative bleeding	0	3	0/2	0.176
Anastomotic leakage	4	2	2/1	0.557
Intestinal obstruction	3	2	1/1	1.000
Impaired liver function	6	4	1/1	1.000
Abdominal distention	0	0	0/0	–

Moreover, we conducted a subgroup analysis based on the number of HIPEC sessions received by patients in the HIPEC group to evaluate whether treatment frequency was associated with a difference in perioperative adverse events. Patients were stratified into subgroups receiving 1, 2, or 3 HIPEC treatments.

The results showed no statistically significant differences in the incidence of any specific adverse events among the three subgroups (all *P* > 0.05). Most adverse events occurred in patients who received only a single HIPEC treatment, likely reflecting the fact that the majority of patients in our cohort underwent one session. No grade 3 adverse events were observed in patients receiving more than one HIPEC treatment. These findings suggest that increasing the frequency of HIPEC does not appear to increase the risk of perioperative complications in patients with locally advanced gastric cancer. No adverse events were found to be uniquely or significantly associated with the HIPEC procedure. While myelosuppression and postoperative bleeding were observed slightly more frequently in the HIPEC group, the differences were not statistically significant. Detailed data are presented in **Table 4**.

**Table 4:** Incidence of perioperative adverse events by number of HIPEC treatments in patients with locally advanced gastric cancer.

Perioperative period Adverse events	1 HIPEC treatment	2 HIPEC treatments	3 HIPEC treatments	*P-*value
Hypoalbuminemia	9	3	0	0.684
Myelosuppression	1	1	0	0.505
Incisional complications	0	0	0	–
Intra-abdominal infection	1	1	0	0.505
Pulmonary infection	5	1	0	0.896
Delayed gastric emptying	0	0	0	–
Anemia	3	1	0	0.895
Postoperative bleeding	3	0	0	0.652
Anastomotic leakage	2	0	0	0.755
Intestinal obstruction	2	0	0	0.755
Impaired liver function	3	1	0	0.895
Abdominal distention	0	0	0	–

## Discussion

Gastric cancer is one of the most common malignant tumors in East Asia, especially in China, where its incidence has been steadily increasing (20). Currently, radical surgical resection is the only curative treatment strategy. Multiple retrospective and prospective studies have shown that radical resection surgery can significantly improve the prognosis of patients (21, 22). Peritoneal recurrence is the most common form of recurrence after radical surgery for gastric cancer, which can lead to intestinal obstruction and malignant ascites (23). This significantly affects the quality of life for patients and shortens overall survival, making it one of the main causes of death among patients (9). However, peritoneal metastasis after radical gastric cancer surgery is almost unavoidable (24, 25). Some studies have indicated that during surgery, the extent of tumor cell spread into the abdominal cavity is higher than during initial exploration, suggesting that surgery can directly promote iatrogenic dissemination of tumor cells, increasing the likelihood of peritoneal metastasis (26). In addition to the increased risk of peritoneal spread due to serosal infiltration of gastric tumors, surgical procedures can also allow cancer cells to infiltrate the abdominal cavity from surgical margins, blood vessels, or lymphatic vessels, ultimately leading to peritoneal metastasis. For patients with advanced gastric cancer with peritoneal metastasis, clinicians employ multimodal treatment strategies to extend survival. A study by Runcong Nie and colleagues shows that conversion surgery can prolong survival in patients with advanced peritoneal metastatic gastric cancer, offering potential survival benefits (27). They also found that palliative gastrectomy combined with first-line chemotherapy can extend survival in patients without multiple distant metastases (28). To address the risk of postoperative peritoneal metastasis in patients with locally advanced gastric cancer, hyperthermic intraperitoneal chemotherapy (HIPEC) has been increasingly explored in recent years. Several randomized controlled trials have reported its potential benefits and safety in this setting (29–33). However, real-world evaluations of the feasibility and safety of combining D2 radical surgery with HIPEC remain limited, particularly in non-randomized, practice-based contexts. Further investigation is needed to assess its clinical applicability and generate hypotheses for future prospective studies.

This is a two-center, retrospective study. The purpose of this study is to explore the feasibility and safety of using hyperthermic intraperitoneal chemotherapy (HIPEC) in patients with locally advanced gastric cancer following D2 radical surgery, and to investigate its potential association with peritoneal recurrence. The results indicate that patients in the HIPEC group had lower peritoneal recurrence rates compared to the surgery-alone group, suggesting that HIPEC may be associated with a reduced risk of peritoneal recurrence and improved prognosis in patients with locally advanced gastric cancer. Independent risk factors for peritoneal recurrence include high Charlson comorbidity index, advanced T stage, vascular invasion, significant intraoperative blood loss, and the absence of postoperative HIPEC treatment. Additionally, the incidence of perioperative adverse events was similar between the two groups, indicating that HIPEC does not increase the risk of such events after D2 radical surgery. These findings support the combination of HIPEC with D2 gastrectomy and highlight the importance of personalized selection of HIPEC candidates for risk stratification.

Previous studies, including those by Yarema et al. and M. Yu Reutovich, have shown that HIPEC significantly reduces peritoneal recurrence and improves disease-free survival compared to surgery alone (30, 34). Our findings align with these studies, showing a lower peritoneal recurrence rate (18.6% vs. 34.9%, *P*=0.0206) in the HIPEC group. The absence of HIPEC was associated with a higher recurrence risk; however, these associations should be interpreted cautiously due to the non-randomized nature of treatment allocation. These results suggest that combining HIPEC with D2 radical surgery may offer clinical benefits in improving patient prognosis for those with locally advanced gastric cancer. In addition to identifying HIPEC treatment as an independent prognostic factor, this study also explored other variables associated with peritoneal recurrence. Consistent with previous findings that tumor infiltration depth, lymph node metastasis, and neural invasion contribute to recurrence risk (35–37). Our analysis further highlighted the prognostic significance of a high Charlson comorbidity index, positive vascular invasion, advanced T stage, higher clinical stage, and substantial intraoperative blood loss.

HIPEC treatment, as an invasive procedure, has drawn attention due to the adverse events associated with this therapeutic approach. Reported adverse events during the perioperative period of HIPEC treatment include leukopenia, peritoneal infection, anastomotic leakage, severe bleeding, acute kidney injury, and intestinal perforation (38–40). These adverse events may also have a detrimental effect on long-term survival outcomes (41). However, with the passage of time, as HIPEC techniques continue to develop and improve, the incidence and mortality rates of related adverse events have gradually decreased (42, 43). Controversy still exists regarding whether there is a difference in the occurrence of complications between patients receiving HIPEC treatment after radical surgery compared to those undergoing radical surgery alone. Felix Merboth et al. reported a retrospective analysis study involving 58 patients, where there was no difference in postoperative complications between the HIPEC group and the radical surgery alone group (both 25%) (44). Jing Zhang et al. reported a retrospective analysis study involving 78 patients with T4 stage gastric cancer, dividing them into single HIPEC group (40 cases) and multiple HIPEC treatment group (38 cases). Both groups experienced mild renal dysfunction, mild hepatic dysfunction, low platelet count, and low white blood cell levels, but the difference in adverse events between the two groups was not statistically significant (*P* > 0.05) (45). Lili Li et al. employed a novel HIPEC protocol using ultrasound-guided puncture needles to establish a closed-loop HIPEC circulation under local anesthesia. Compared to the traditional method, using smaller diameter needles without the need for catheter insertion made the HIPEC treatment process more tolerable. The study results showed no significant difference in adverse events between the HIPEC treatment group and the control group. Mild procedure-related side effects observed mainly included self-resolving puncture site pain and mild peritonitis (29). In this study, there was no difference in the occurrence of perioperative adverse events between the HIPEC group and the radical surgery alone group (*P* > 0.05), and there was no statistically significant difference in the occurrence of adverse events between different frequencies of HIPEC treatment groups (*P* > 0.05). Overall, adjunctive HIPEC therapy appears to be effective and safe.

Despite advances in HIPEC, several key challenges persist. First, the indications for HIPEC in gastric cancer and the criteria for selecting patients who will benefit remain unclear. Although the Peritoneal Cancer Index (PCI) is widely used and validated as an independent prognostic factor for peritoneal metastasis in gastric cancer, its clinical utility is debated (46, 47). For example, up to 40% of patients with negative preoperative imaging have peritoneal metastases detected during diagnostic laparoscopy (48). Moreover, even if laparoscopy reveals no macroscopic disease, up to 13% of patients have positive peritoneal cytology and later develop peritoneal recurrence (49). Which clinical or molecular subgroups benefit most from HIPEC is also unknown, partly because clinicians often lack data on key biomarkers (e.g., PD-L1 expression, tumor mutational burden, microsatellite stability, CLDN18.2 status). Notably, high PD-L1 expression correlates with improved survival in gastric cancer, particularly in patients with peritoneal metastases. In contrast, CLDN18.2 positivity and HER2 overexpression are linked to poorer outcomes (50–53). Moreover, the distribution, metabolism, and mechanism of action of chemotherapeutic agents during HIPEC within the peritoneum and tumor tissue remain unclear. *In vitro* studies have shown that miR−218 enhances the sensitivity of gastric cancer cells to cisplatin, and HIPEC has been reported to significantly upregulate miR−218 levels (54). Additionally, hyperthermia markedly increases the cytotoxicity of 5−fluorouracil (5−FU) in gastric cancer cells, induces PARP and caspase−3 cleavage to promote apoptosis, and significantly upregulates PD−L1 expression on the tumor cell surface (55). Furthermore, HIPEC protocols lack standardization. Published reports reveal significant variability in drug selection, dosing, perfusion temperature, and duration across centers, hindering outcome comparisons (30, 56–59). Therefore, multicenter randomized controlled trials are needed to systematically evaluate these parameters and establish optimized, standardized protocols. Finally, patients receiving HIPEC after D2 gastrectomy remain at risk for complications such as leukopenia, peritoneal infection, severe hemorrhage, renal dysfunction, and intestinal perforation. The relationship between hyperthermic perfusion and postoperative complications remains controversial (45, 60–62). Optimizing perfusion temperature and duration, co-administering protective agents, and refining perioperative management guidelines may reduce these complication rates (63–65). Over the next few years, the HIPEC field is poised for major advances in multiple areas.

First, multicenter randomized trials will systematically compare key variables—drug selection, dosage, perfusion temperature, and duration—to establish standardized global protocols. Second, integrating molecular and imaging biomarkers (e.g., genomic alterations, protein expression, and preoperative imaging) will enable precise patient selection and risk stratification for HIPEC. Third, new heat−stable chemotherapeutics, nanocarriers, and liposomal systems will improve intraperitoneal drug delivery and tumor penetration while minimizing toxicity to healthy tissues. Fourth, combining HIPEC with immunotherapies—such as checkpoint inhibitors, CAR−T cells, or cancer vaccines—may enhance tumor immunogenicity, overcome “cold” tumor resistance, and increase therapeutic efficacy. Finally, large-scale clinical studies will build integrated clinical and genomic HIPEC databases. Machine learning models can then predict treatment outcomes and complication risks, supporting personalized HIPEC strategies.

This study has several limitations. First, its retrospective design and relatively short follow-up durations may affect the accuracy of recurrence assessment. Second, patients who did not receive standard adjuvant chemotherapy were excluded, which may introduce allocation bias. Third, the treatment allocation was non-randomized and influenced by real-world factors such as economic constraints, which may lead to selection bias and limit the ability to draw causal conclusions. Therefore, the observed associations between HIPEC and clinical outcomes should be interpreted with caution, and not as definitive evidence of efficacy. Lastly, although this was a dual-center study, the overall sample size was relatively limited, which may affect the generalizability of the findings. Future large-scale, multicenter randomized controlled trials are needed to validate these findings and support causal interpretation.

## Conclusion

The study results suggest that adjuvant HIPEC following D2 radical surgery may be associated with a reduced risk of peritoneal recurrence in patients with locally advanced gastric cancer. In our cohort, combining D2 radical surgery with HIPEC—and performing multiple HIPEC sessions—was not associated with an increased risk of perioperative adverse events. However, some patients did not undergo HIPEC due to economic constraints, resulting in non-random treatment allocation and introducing potential selection bias. This form of bias cannot be addressed through statistical adjustment and may affect the interpretation of causality. Therefore, the findings should be interpreted as observational associations rather than definitive treatment effects. Standardized protocols for patient selection and technical parameters (perfusate type, duration, flow rates, temperature) are needed, and future studies using propensity score–based methods and large multicenter randomized controlled trials are essential to further evaluate the efficacy and safety of HIPEC.

## Data Availability

The raw data supporting the conclusions of this article will be made available by the authors, without undue reservation.
